# PMLocMSCAM: Predicting miRNA Subcellular Localisations by miRNA Similarities and Cross‐Attention Mechanism

**DOI:** 10.1049/syb2.70023

**Published:** 2025-06-08

**Authors:** Jipu Jiang, Cheng Yan

**Affiliations:** ^1^ School of Informatics Hunan University of Chinese Medicine Changsha China

**Keywords:** bioinformatics, biology computing, cross‐attention mechanism, miRNA subcellular localisation

## Abstract

Many studies have shown that microRNAs (miRNAs) play key roles in some important processes and human complicated diseases. In addition, they also have specific physiological roles at different cellular sites. Therefore, identifying their subcellular localisation is very urgent to systemically understand their physiological functions. In this study, we propose a computational method, called PMLocMSCAM, to predict miRNA subcellular localisation based on miRNA similarities and cross‐attention mechanism. PMLocMSCAM implements a multimodal integration framework that systematically processes miRNA sequence data, miRNA‐mRNA association networks with mRNA subcellular localisation annotations, miRNA‐disease associations, and miRNA‐drug association networks. The architecture initiates with intrinsic feature extraction through Smith‐Waterman alignment for sequence similarity computation and disease ontology‐based functional similarity derivation. Subsequent heterogeneous network embedding employs Node2vec for topological feature learning across three interaction modalities (miRNA‐disease, miRNA‐drug, and miRNA‐mRNA networks), enhanced by hypergraph convolution to capture higher‐order relationships through incidence matrix decomposition. Localisation‐specific patterns are propagated via miRNA‐mRNA interaction weights, culminating in a multi‐head attention mechanism that dynamically fuses five feature matrices—miRNA sequence features, miRNA‐disease association features, miRNA‐drug association features, miRNA‐mRNA association features, and miRNA‐mRNA localisation features. These integrated representations are processed through residual‐connected multilayer perceptrons to generate probabilistic predictions across seven subcellular compartments, establishing an end‐to‐end computational paradigm for multimodal miRNA localisation analysis. In order to assess the prediction performance of our method and compare it with other miRNA subcellular localisation computational methods, we conduct 10‐fold cross validation (10‐CV) and independent test dataset. The AUC (area of receiver operating characteristic curve) and AUPR (area of precision‐recall curve) are used as metrics. The experiment results show that the average AUC and AUPR values exceed 0.9182 and 0.8487 on 10‐CV, respectively. The AUC and AUPR values also reach 0.9157 and 0.8469 on independent test dataset, respectively. It is superior with compared methods. The ablation experiment results also further that PMLocMSCAM can effective predict miRNA subcellular localisations and provide help to understand their physiological functions.

## Graphical abstract

Identifying miRNA subcellular localisation is very urgent to systemically understand their physiological functions. In this study, we propose a computational method, called PMLocMSCAM, to predict miRNA subcellular localisation based on miRNA similarities and cross‐attention mechanism. The experiment results show that PMLocMSCAM can effective predict miRNA subcellular localisations and provide help to understand their physiological functions.

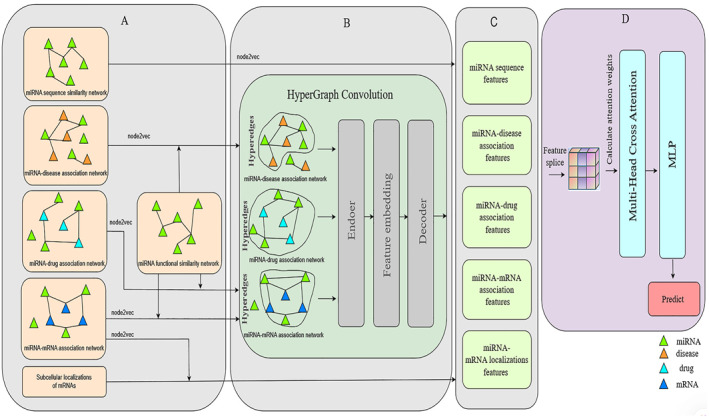

## 1 Introduction

MicroRNAs (miRNAs) are a group of noncoding RNAs and 22 nucleotides long, and they take part in some important biological processes [1], such as cell development, division, metabolism, differentiation and death [2]. Many studies also have shown that the abnormal expression or dysfunction of miRNAs is closely associated with many complex human diseases, such as cancer [3, 4, 5]. The expression of preoperative levels of five faecal miRNAs was significantly higher in colorectal cancer (CRC) patients compared to controls and significantly decreased after curative surgery [3]. They can be considered as an appealing opportunity for CRC secondary prevention. MiR‐101‐2, miR‐125b‐2 and miR‐451a were found to be down‐regulated in the primary gastric cancer (GC) tissues, and they can function as potential tumour suppressors in GC [4]. A significant overexpression of miR‐142‐3p and miR‐155 was observed in gastric MALT lymphoma, and down‐regulation of miR‐203 was also observed in gastric MALT lymphoma [5]. Their expression levels can be the helpful biomarkers of gastric MALT lymphomas. In addition, since not every protein can be targeted by small molecular, recent studies have illustrated that miRNAs can be new targets of drugs [6, 7, 8, 9]. The variations in miRNA profiling of patients can be a major cause of individual differences in drug sensitivity or resistance [6]. Overexpressed miRNAs can downregulate genes with protein products necessary for drug efficacy and insufficient miRNA expression can also upregulate genes with protein products inhibiting drug function [7].

Recent studies have also illustrated that miRNA subcellular localisations can influence their functionality, as it also indicates that different subcellular locations can occur in different gene expression [10, 11]. The miRNAs subcellular localisation also can influences the cellular processes involved in development, proliferation and digestion [12]. For example, miRNAs localised to nucleus participate in mitosis or gene expression regulation [13]. MiRNAs have been known to function as a component of miRNA‐induced silencing complex (miRISC) in the cytoplasm, and which can be translocated back to the nucleus [14, 15]. In addition, they have also been discovered in other cellular compartments, such as exosome [16], nucleolus [17], nucleus [18], extracellular vesicle [19], mitochondrionv [20] and microvesicle [21]. The exosomes were involved in cell‐to‐cell communication, and the exosome miRNAs can be functionally transferred to recipient cells [16]. The microvesicles also were the important mediators of cell‐to‐cell communication [20]. In cancer cells, studies have also shown that the mitochondria miRNAs can bind to mRNAs encoded by mtDNAs, enabling regulation of mitochondria‐related functions [20]. Therefore, in order to systemically understand the physiological functions of miRNA, we need to identify the miRNA subcellular localisations. Some computational methods have proposed to predict miRNA subcellular localisations since traditional experimental‐based methods requires significant experimental time and cost. miRLocator was a prediction method of miRNAs subcellular locations, which is an attention‐based encoder‐decoder model [22]. Based on the miRNA's GO annotations, Yang et al. proposed a computational method to predict miRNA subcellular localisations by calculating the functional similarity for miRNAs [23]. MirLocPredictor was also a sequence‐based miRNA subcellular localisations prediction method [24]. It applied kmerPR2vec model to obtain the miRNA feature representation, which is a fusion of positional information of k‐mers with randomly initialised neural embeddings. Based on the principal component scores of thermodynamic, structural properties and pseudo compositions of dinucleotides, Meher et al. also proposed a computational method for identifying miRNA subcellular localisations [25]. BioSeq‐Analysis2.0, iLearnPlus and Math‐Feature were the comprehensive platform for nucleic acid and protein sequence analysis and prediction, and the extracted miRNA feature representations were applied in predicting miRNA subcellular localisations [26, 27, 28]. To categorise functionally similar locations for the precise and instructive prediction of miRNA subcellular location, iLoc‐miRNA was proposed by a deep bidirectional long short‐term memory with the multi‐head self‐attention mechanism [29]. MiRLoc was a miRNA subcellular locations prediction method, the main feature of this method is that it incorporates miRNA‐mRNA associations [30]. DAmiRLocGNet was a miRNA subcellular localisation prediction method based on graph convolutional network (GCN) and autoencoder (AE) [31]. The main contribution of DAmiRLocGNet is that it extracted from miRNA feature representation from miRNA‐disease association network and miRNA sequence similarity network by GCN and AE, respectively. Liang et al. proposed a deep learning‐based method, called MGFmiRNAloc, to predict miRNA subcellular localisation based on GCN and the channel attention and spatial attention mechanisms (CBAM) [32]. In addition, based on miRNA sequence, miRNA functional similarity, miRNA‐disease, miRNA‐drug and miRNA‐mRNA associations, PMiSLocMF was also proposed to predict miRNA subcellular localisation by node2vec and graph attention auto‐encoder (GATE) [33].

Although these computational methods have obtained promising results in identifying miRNA subcellular localisations, the prediction performances also should be further improved. In this study, we also proposed a new computational method (called PMLocMSCAM) to predict miRNA subcellular localisations. PMLocMSCAM implements a multimodal integration framework that systematically processes miRNA sequence data, miRNA‐mRNA association networks with mRNA subcellular localisation annotations, miRNA‐disease associations, and miRNA‐drug association networks. The architecture initiates with intrinsic feature extraction through Smith‐Waterman alignment for sequence similarity computation and disease ontology‐based functional similarity derivation. Subsequent heterogeneous network embedding employs Node2vec for topological feature learning across three interaction modalities (miRNA‐disease, miRNA‐drug, and miRNA‐mRNA networks), enhanced by hypergraph convolution to capture higher‐order relationships through incidence matrix decomposition. Localisation‐specific patterns are propagated via miRNA‐mRNA interaction weights, culminating in a multi‐head attention mechanism that dynamically fuses five feature matrices—miRNA sequence features, miRNA‐disease association features, miRNA‐drug association features, miRNA‐mRNA association features, and miRNA‐mRNA localisation features. These integrated representations are processed through residual‐connected multilayer perceptrons to generate probabilistic predictions across seven subcellular compartments, establishing an end‐to‐end computational paradigm for multimodal miRNA localisation analysis. We conduct the 10‐fold cross validation (10‐CV) and independent test dataset to assess the prediction performance of our method. The AUC (area of receiver operating characteristic curve) and AUPR (area of precision‐recall curve) are used as metrics, which also were used in previous studies. We also compare our method to other computational methods for predicting miRNA subcellular localisations. The experiment results of benchmark dataset show that our method obtains best prediction performances. In 10‐fold cross‐validation, the AUC and AUPR values achieve 0.9182 and 0.8487, respectively. On independent test dataset, the AUC and AUPR values also reach 0.9157 and 0.8469, respectively. The ablation experiments and analysis of feature representation also illustrate that PMLocMSCAM can effectively identify the miRNA subcellular localisations.

## 2 Materials and Methods

### 2.1 Dataset

In this study, the benchmark dataset is downloaded from RNALocate database (version 2.0) [34]. It includes 1041 miRNAs and also widely used in previous studies [30, 31]. These miRNAs are classified into seven subcellular localisations (cytoplasm, exosome, nucleolus, nucleus, extracellular vesicle, microvesicle, and mitochondrion) and each localisation contains more than 50 miRNAs. In addition, the miRNA sequences are downloaded from miRBase database which is the primary online repository for all microRNA sequences and annotation and consists of the pre‐miRNA sequences and mature‐miRNA sequences of human, rat and mouse [35]. We obtain the miRNA‐disease associations from HMDD v 3.2 which is a comprehensive database of miRNA‐disease associations [36]. After projecting process, we obtain 1041 miRNAs, 640 diseases and 15,547 associations between them. To construct the miRNA‐mRNA association network, we obtain the miRNA‐mRNA associations from database miRTarBase which has accumulated > 13,404 validated miRNA‐target interactions from 11,021 articles from manual curations [37]. After projecting process, the obtained miRNA‐mRNA association network includes 1041 miRNAs, 2836 mRNAs and 8254 associations between them. Furthermore, in order to further validate the prediction performance of our method in real application, we also download the independent test dataset from previous study, which includes 41 miRNAs. The miRNAs of independent test data also can belong to multiple subcellular localisations.

### 2.2 Method

**FIGURE 1:**
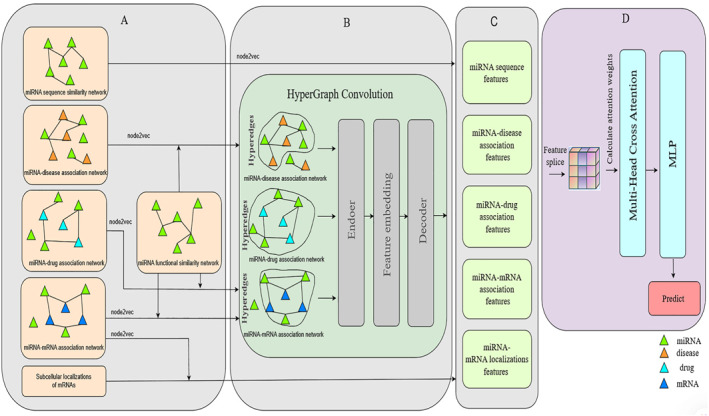
Overview of PMLocMSCAM.The PMLocMSCAM framework is designed with four key stages: similarity calculation and initial associated network, initial feature, feature extraction, and prediction subcellular localisation. (A) Calculating similarity and constructing initial association network, (B) obtaining original feature, (C) extracting final feature, (D) predicting miRNA subcellular localisation.

We propose a model for predicting miRNA subcellular localisations based on miRNA similarities and the cross‐attention mechanism. As shown in Figure 1, PMLocMSCAM comprehensively integrates miRNA sequence information, miRNA‐mRNA associations and mRNA subcellular localisation data, miRNA‐disease associations, and miRNA‐drug interactions to accurately predict miRNA subcellular localisation. First, miRNA sequence similarity and functional similarity are calculated based on miRNA sequences and miRNA‐disease associations, respectively, to extract sequence features. Then, miRNA functional similarity is integrated with miRNA‐disease association networks, miRNA‐drug interaction networks, and miRNA‐mRNA association networks. Node2vec and Hypergraph Convolution are employed for deep feature extraction to construct miRNA‐associated network features. Additionally, miRNA‐mRNA associations and mRNA subcellular localisation data are incorporated to extract miRNA localisation features. Finally, a multi‐head attention mechanism and Multilayer Perceptron (MLP) are utilised to effectively integrate these five types of features, generating high‐dimensional, information‐rich miRNA representations for subcellular localisation prediction.

### 2.3 Constructing miRNA‐mRNA Association Network and mRNA Subcellular Localisations

In this study, we also integrate miRNA‐mRNA associations to obtain miRNA feature representation by considering the contribution of miRNA‐mRNA association to prediction miRNA subcellular localisation. mRNAs are a widely studied type of RNAs, such as the research and development of mRNA cancer vaccines through the application of personalised cancer antigens. Recent studies also prove that the miRNA‐mRNA association network is the effective biological network to help miRNA subcellular localisation. In this study, we also apply the miRNA‐mRNA association network which includes 1041 miRNAs, 2836 mRNAs and 8254 associations between them [37]. In addition, by considering the subcellular localisations of mRNAs also have associated the miRNA subcellular localisations, we also apply the mRNA subcellular localisations. For 2836 mRNAs of miRNA‐mRNA association network, they belong to seven types of subcellular localisation (cytoplasm, exosome, nucleolus, nucleus, extracellular vesicle, microvesicle and mitochondrion). Some mRNAs have multiple subcellular localisations, such as 877 mRNAs have four localisations (cytoplasm, exosome, nucleolus and nucleus).

### 2.4 Constructing miRNA‐Disease Association Network and miRNA‐Drug Association Network

In addition to miRNA‐mRNA association network, the miRNA‐disease associations also are employed in this study to extract miRNA feature representations. In previous study [31], the miRNA‐disease associations are also used to predict miRNA subcellular localisations. In this study, the miRNA‐disease associations of benchmark dataset are downloaded from database HMDD v3.2 [36], which includes 1041 miRNAs, 640 diseases and 15,547 associations between them. Based on these miRNAs, diseases and associations between them, the miRNA‐disease association network is constructed.

Similar to disease mechanisms, pharmacological agents demonstrate established miRNA interactions. Therapeutic compounds linked to specific miRNAs may assist in predicting their intracellular localisation patterns. Our computational framework integrated this pharmacological dimension into miRNA localisation prediction, utilising miRNA‐drug association data from ncDR, a specialised database documenting noncoding RNA interactions with drug resistance mechanisms. From 1041 filtered miRNAs, we extracted 3305 validated associations involving 130 therapeutic agents. These interactions formed a bipartite network (Nmi‐dr) connecting miRNA nodes with drug nodes through weighted edges, creating a pharmacological topology for machine learning applications.

### 2.5 Constructing the miRNA Similarity Network

In this study, we construct miRNA sequence similarity, miRNA functional similarity and miRNA GIP kernel similarity. In addition, by considering the phenomenon that the miRNA functional similarity value is zero, we fuse miRNA functional similarity and miRNA GIP kernel similarity. Therefore, we first calculate these similarities as follows.

#### 2.5.1 Calculating miRNA Sequence Similarity

The sequence is the most basic information for miRNA. In this study, by considering the successful application of sequence similarity of miRNA, we calculate the miRNA sequence similarity by Smith–Waterman algorithm. For miRNA m1 and m2, the sequence similarity SW(m1,m2) of them can be calculated as follows:

(1) $$SW\left(m1,m2\right)=\frac{sp\left(m1,m2\right)}{\sqrt{sp\left(m1,m1\right)•sp\left(m2,m2\right)}},$$

In which sp(m1,m2) represents the local alignment score of two sequences. In addition, based on the miRNA sequence similarities also applied in previous studies [31, 33], we download directly it from DAmiRLocGNet.

#### 2.5.2 Calculating miRNA Functional Similarity

Furthermore, we also calculate the miRNA functional similarity. The miRNA similarity is computed by the miRNA‐disease associations and disease sematic information. Therefore, we first denote the disease to a direct acyclic graph (DAG) based on its medical subject heading (MESH). The nodes of a DAG include it and all its ancestor nodes. The edges of this DAG include all edges between all nodes of it. So, each disease A can be represented a subgraph and the contribution ${SV}_{A}\left(t\right)$ of a disease *t* can be calculated as follows:

(2) $$SV_{A}\left(t\right)=\left\{\begin{array}{cc} 1\; & \;\text{if}\;t=A\\ \Delta^{k}\; & \;\text{if}\;t=the\;smallest\;K\;layer,\;a\;ancector\;node\;of\;A, \end{array}\right.$$

In which Δ is the hyperparameter and is used to control contribution between disease and its direct ancestor disease. We also set it to be 0.5 according previous studies [31, 33]. Therefore, we can obtain the sematic value $Sem\left(A\right)$ of disease A as follows:

(3) $$Sem\left(A\right)=\sum_{t\in T_{A}}{SV}_{A}\left(t\right),$$

In which $T_{A}$ is the set of all parent diseases nodes of A and itself. Therefore, the sematic similarity $D_{SS}\left(A,B\right)$ of diseases A and B is can be calculated as follows:

(4) $$D_{SS}\left(A,B\right)=\frac{\sum_{t\in T_{A}\cap T_{B}}\left({SV}_{A}\left(t\right)+{SV}_{B}\left(t\right)\right)}{Sem\left(A\right)+Sem\left(B\right)}.$$

Based on the sematic similarities among diseases, we can calculate the sematic similarity value of disease and disease set. For disease ${dt}_{2j}$ and disease set $DT_{1}$, their sematic similarity value $S\left({dt}_{2j},DT_{1}\right)$ can be calculated as follows:

(5)

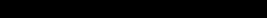

where n2 is the number of disease set $DT_{1}$. Therefore, according to the disease sematic information and miRNA‐disease associations, we can calculate the miRNA functional similarity. For miRNAs m1 and m2, the functional similarity between them can be calculated as follows:

(6) $$FS\left(m_{1},m_{2}\right)=\frac{\sum_{1\leq i\leq n_{1}}S\left({dt}_{1i},DT_{2}\right)+\sum_{1\leq j\leq n_{2}}S\left({dt}_{2j},DT_{1}\right)}{n_{1}+n_{2}}.$$

#### 2.5.3 Calculating miRNA Gaussian Interaction Profile (GIP) Kernel Similarity

In addition to miRNA sequence similarity and functional similarity, we also compute the Gaussian Interaction Profile (GIP) kernel similarity of miRNAs, which leverages the topological structure of the known miRNA‐disease association network. The core idea behind GIP similarity is that miRNAs associated with similar diseases tend to be similar to each other, and vice versa. In this study, the miRNA‐disease association network comprises 1041 miRNAs, 640 diseases and 15,547 known associations. Let the adjacency matrix $Y\in R^{N_{m}\times N_{d}}$ represent these known associations, where $N_{m}$ and $N_{d}$ denote the number of miRNAs and diseases, respectively. Each row $y_{m_{i}}=\left[y_{i1},y_{i2},\text{…},y_{iN_{d}}\right]$ represents the association profile of miRNA $m_{i}$, where $y_{ij}=1$ indicates an association with disease $d_{j}$, and 0 otherwise. The GIP kernel similarity between two miRNAs $m_{1}$ and $m_{2}$ is defined as follows:

(7) $$GIP\left(m_{1},m_{2}\right)=\exp\left(-{\gamma_{m}\left|\left|ym_{1}-ym_{2}\right|\right|}^{2}\right)$$

(8) $$\gamma_{m}=\gamma_{m}^{\prime }/\left(\frac{1}{N_{m}}\sum_{i=1}^{N_{m}}{\left|\left|ym_{i}\right|\right|}^{2}\right),$$

where $\gamma_{m}^{\prime }$ is a kernel bandwidth hyperparameter set to 1, as adopted in previous studies [31, 38].

#### 2.5.4 Integrating miRNA Functional and GIP Kernel Similarities

By considering the phenomenon that the miRNA functional similarity value is zero, we integrate the miRNA functional similarity and GIP kernel similarity by as follows:

(9) $$FS\left(m_{1},m_{2}\right)=\left\{\begin{array}{cc} FS\left(m_{1},m_{2}\right)\; & \;\text{if}\;FS\left(m_{1},m_{2}\right)>0\\ GIP\left(m_{1},m_{2}\right)\; & \;\text{if}\;otherwise.\; \end{array}\right.$$

### 2.6 Obtaining the miRNA Origin Feature Representation

#### 2.6.1 Node2Vec

Node2Vec is an unsupervised network representation learning method that embeds nodes into a low‐dimensional vector space by generating random walk sequences in graphs to capture both structural properties and semantic information of nodes. The Node2Vec method constructs a miRNA‐disease association graph and performs random walks on it to capture structural information of miRNAs within the network. First, miRNAs and diseases are treated as nodes, with edges added based on the miRNA‐disease association matrix to form an undirected graph. Subsequently, Node2Vec executes random walks on this graph, where each miRNA establishes contextual relationships through generated walk paths, and employs the Skip‐gram model to train low‐dimensional vector representations. By combining strategies of Depth‐First Search (DFS) and Breadth‐First Search (BFS), Node2Vec can learn both local neighbourhood information and global network structure, enabling the extracted features to characterise the topological positions of miRNAs in the network and their association patterns with diseases. Ultimately, the trained 128‐dimensional miRNA feature vectors are preserved for subsequent tasks.

#### 2.6.2 Hypergraph Convolution

All three features we constructed used hypergraph convolution, including miRNA‐drug association features, miRNA‐mRNA association features, and miRNA‐disease association features. Taking miRNA‐drug association modelling as an example, hypergraphs constructed using miRNA functional similarity networks and miRNA‐drug association feature matrices can more comprehensively characterise the multivariate relationships between miRNAs and drugs. Unlike traditional graph structures, hypergraphs allow hyperedges to connect multiple miRNAs, thereby naturally expressing functional associations among miRNAs and higher‐order interaction patterns where miRNAs jointly act on drugs. During hypergraph construction, miRNAs are first treated as nodes, while hyperedges are established based on two types of information: 1) functional similarity between miRNAs, and 2) miRNA‐drug association network. In the functional similarity network, miRNAs with high similarity exhibit stronger connection weights, suggesting they may share analogous biological functions. Therefore, by setting similarity thresholds, highly similar miRNAs can be grouped into the same hyperedge, enabling the model to learn mutual influences among miRNAs at the functional level. On the other hand, the miRNA‐drug association matrix provides direct relationship information between miRNAs and drugs. When multiple miRNAs are associated with the same drug, these miRNAs can be jointly connected through a hyperedge corresponding to that drug. This modelling approach not only captures direct miRNA‐drug interactions but also implicitly introduces interconnections between miRNAs. For instance, certain miRNAs without direct functional similarity may co‐regulate the same drug, implying potential indirect associations through the drug as a bridge. Such relationships are challenging to represent in traditional graphs but can be naturally characterised by hyperedges in hypergraphs. The hypergraph convolution formula is as follows:

(10) $$\hat{A}=D^{-1/2}A_{\text{HyperGraph}}D^{-1/2},$$

(11) $$H^{\left(l+1\right)}=\sigma \left(\hat{A}H^{\left(l\right)}W^{\left(l\right)}\right).$$

In the process of Hypergraph Convolution, node feature representations are aggregated through hyperedges. Unlike traditional Graph Convolutional Networks (GCNs), which only consider pairwise node relationships, HGCN captures interaction patterns among multiple miRNAs via hyperedges. This allows miRNA embeddings to fuse not only their intrinsic features but also information from all related miRNAs and drugs. Through multi‐layer propagation, the final embeddings of miRNAs integrate both local and global information, enabling the model to more accurately learn latent functional relationships among miRNAs and miRNA‐drug interaction characteristics. The miRNA‐mRNA association features and miRNA‐disease association features follow the same principles as described above.

#### 2.6.3 Obtaining the miRNA Final Feature Representation by Cross‐Attention Mechanismn

In the miRNA multi‐modal feature fusion process, we employ a cross‐attention mechanism to extract the final feature representation, fully leveraging the relationships among sequence features, mRNA co‐localisation features, mRNA network features, and disease features. First, we use fully connected layers to map each input feature to the same dimension for subsequent fusion calculations:

(12) $$\begin{array}{c} H_{\mathrm{seq}}=\sigma \left(W_{\mathrm{seq}}X_{\mathrm{seq}}+b_{\mathrm{seq}}\right),\\ H_{co\text{\_}loc}=\sigma \left(W_{co\text{\_}loc}X_{co\text{\_}loc}+b_{co\text{\_}loc}\right),\\ H_{\mathrm{net}}=\sigma \left(W_{\mathrm{net}}X_{\mathrm{net}}+b_{\mathrm{net}}\right),\\ H_{\mathrm{dis}}=\sigma \left(W_{\mathrm{dis}}X_{\mathrm{dis}}+b_{\mathrm{dis}}\right), \end{array}$$

where W and b are trainable weights and biases, and ReLU denotes the activation function. Subsequently, we concatenate features from different sources to form a complete high‐dimensional feature representation:

(13) $$H=\left[H_{\mathrm{seq}},H_{co\text{\_}loc},H_{\mathrm{net}},H_{\mathrm{dis}}\right].$$

To emphasise important features, we use a fully connected layer to generate attention weights and normalise them via softmax to ensure the sum of all feature weights equals 1:

(14) $$A=softmax\left(W_{\mathrm{att}}H+b_{\mathrm{att}}\right).$$

We then perform a weighted summation of the features to select the most representative feature information:

(15) $$H_{\mathrm{selected}}=A⊙H.$$

Next, we utilise the multi‐head cross‐attention mechanism to further explore deep interaction relationships among features. Specifically, we use the selected features as Query (Q), the original concatenated features as Key (K) and Value (V), and compute attention scores:

(16) $$Attention\left(Q,K,V\right)=softmax\left(\frac{QK^{T}}{\sqrt{d_{k}}}\right)V,$$

where $d_{k}$ is a scaling factor for the feature dimension. By employing the multi‐head attention mechanism, we concatenate the results from multiple attention heads and integrate them through a linear transformation:

(17) $$MultiHead\left(Q,K,V\right)=Concat\left({head}_{1},{head}_{2},\text{…},{head}_{h}\right)W_{O},$$

where $W_{0}$ is a trainable transformation matrix, and headiheadi represents the computation result of the *i*th attention head. Finally, we apply residual connections and layer normalisation to maintain information flow and enhance training stability:

(18) $$H_{\mathrm{final}}=LayerNorm\left(H+MultiHead\left(H_{\mathrm{selected}},H,H\right)\right).$$

The fused miRNA feature representation $H_{\mathrm{final}}$ is used for subsequent subcellular localisation prediction tasks.

### 2.7 Experimental Settings

To ensure the effective training and stable convergence of the proposed PMLocMSCAM model, a standardised and well‐validated experimental configuration is adopted. The model is implemented using the PyTorch deep learning framework and trained with the Adam optimiser which is well‐regarded for its adaptive learning rate and superior performance on complex nonconvex optimisation tasks. The initial learning rate is set in the range of 0.001–0.005 with an interval 0.001. According to general experimental settings [39], we set the batch size to 64. We conduct experiments with 50, 100 and 200 training epochs. Based on experiment results, training for 100 epochs is selected. An early stopping strategy is implemented, where training is halted if the validation loss fails to improve for 50 consecutive epochs to prevent overfitting. Model weights are initialised using the Xavier (Glorot) initialisation method to maintain stable activation variance across layers, facilitating faster convergence.

In the model architecture design, based on the distribution characteristics of the data, the input features of miRNA, disease, and drug are embedded into 128‐dimensional vectors. For the hypergraph convolution layers, we evaluate architectures with 1–4 layers, and find that using 3 layers yields the optimal results. Among all model parameters, those associated with the hypergraph convolution layers have the greatest impact on model performance fluctuations. Detailed analysis is provided in the subsection ‘Ablation Experiments and Parameter Analysis’. To mitigate overfitting, a dropout rate of 0.3 is applied after each attention and feed‐forward layer. The ReLU activation function is used throughout the network to enhance nonlinear representation capability.

## 3 Predicting miRNA Subcellular Localisations

After obtaining the final miRNA feature representation, we employ a Multilayer Perceptron (MLP) for multi‐label prediction of miRNA subcellular localisation. Since a single miRNA may reside in multiple subcellular locations simultaneously, this task is a multi‐label classification problem rather than single‐label classification. To accommodate this, the output layer uses multiple neurons, each corresponding to a subcellular location, with the sigmoid function outputting the probability for each category:

(19) $${\hat{y}}_{ij}=\sigma \left(W_{\mathrm{out}}H_{\mathrm{final}}+b_{\mathrm{out}}\right),$$

where $W_{\mathrm{out}}$ and $b_{\mathrm{out}}$ are trainable parameters of the output layer, and σ is the sigmoid activation function, ensuring output values between 0 and 1. To optimise the model, we adopt Binary Cross‐Entropy (BCE) as the loss function:

(20) $$L=-\frac{1}{N}\sum_{i=1}^{N}\sum_{j=1}^{C}\left[y_{ij}\;\log{\hat{y}}_{ij}+\left(1-y_{ij}\right)\log\left(1-{\hat{y}}_{ij}\right)\right],$$

where N is the number of samples, C is the number of subcellular location categories, $y_{ij}$ is the true label of the *i*th sample for the *j*th class, and ${\hat{y}}_{ij}$ is the predicted probability. As this task involves multi‐category prediction, we use the Adam optimiser for gradient updates to accelerate convergence. During model training, we apply 10‐fold cross‐validation to fully utilise the data and implement an early stopping mechanism to prevent overfitting. After training, we evaluate model performance using AUC (Area Under the ROC Curve) and AUPR (Average Precision) to measure classification capability across categories and overall recall, ensuring the final miRNA prediction results exhibit strong discriminative power and practical value.

## 4 Results

### 4.1 10‐Fold Cross‐Validation

**FIGURE 2:**
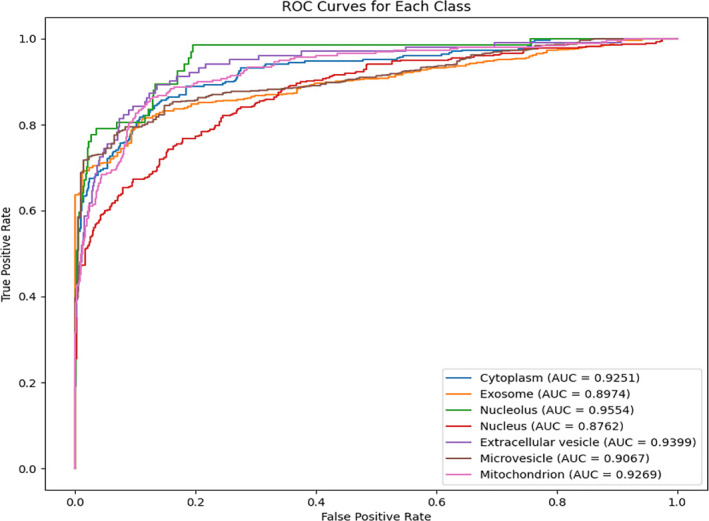
The AUC value of the model PMLocMSCAM.

**FIGURE 3:**
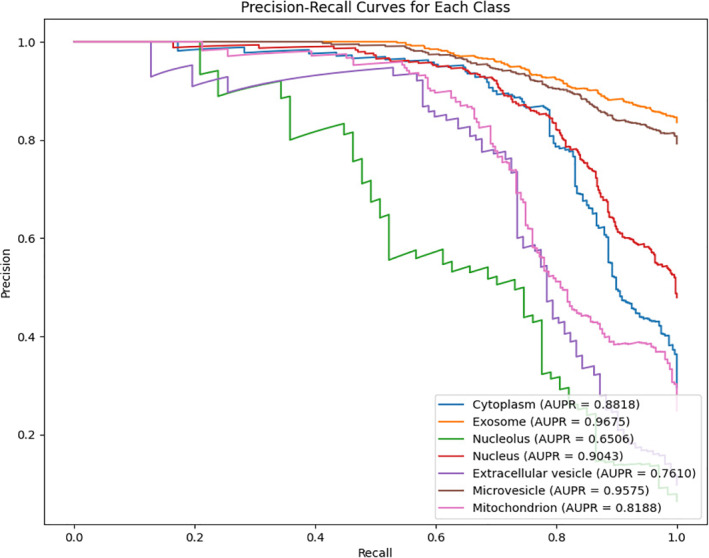
The AUPR value of the model PMLocMSCAM.

After performing 10‐fold cross‐validation (10‐CV), we plotted the ROC curves and Precision‐Recall (PR) curves for the model across all categories. Figure 2 displays the ROC curves for each category with annotated AUC values, demonstrating strong overall performance and indicating the model's robust classification capability. Figure 3 shows the PR curves for all categories, illustrating the precision‐recall trade‐off at different recall levels, along with AUPR (Area Under the PR Curve) values. Overall, the model exhibits excellent predictive performance across most categories. Aggregating all categories, the model achieved an average AUC of 0.9182 and an average AUPR of 0.8487, further validating its effectiveness and robustness in multi‐class tasks.

To further verify our model's performance, we compare it with other models, as shown in Tables 1 and 2. Table 1 presents the comparison of AUC metrics with other models. The PMLocMSCAM model outperforms the PMiSLocMF model in AUC for most categories, particularly in the ‘Extracellular vesicle’ category, where PMLocMSCAM achieves an AUC of 0.9399, significantly higher than PMiSLocMF's 0.8574. This notable improvement can be attributed to the model's multi‐scale convolutional design and cross‐attention mechanism, which enable it to effectively distinguish between highly overlapping subtypes (e.g., exosomes and microvesicles). These subtypes often share similar sequence features and secretion patterns, making fine‐grained classification particularly challenging for single‐scale or shallow models. Additionally, the average AUC of PMLocMSCAM (0.9182) surpasses that of PMiSLocMF (0.9033), demonstrating its superior overall classification capability and generalisation.

**TABLE 1:** AUC comparison of PMLocMSCAM with other models.

Subcellular localisation	MiRLoc	MirLocPredictor	DAmiRLocGNet	PMiSLocMF	PMLocMSCAM(ours)
Cytoplasm	0.8366	0.5741	0.8606	0.8909	**0.9251**
Exosome	0.7395	0.5842	0.7051	**0.9513**	0.8974
Nucleolus	0.9085	0.5286	0.9289	0.9267	**0.9554**
Nucleus	0.7765	0.6752	0.7960	**0.8764**	0.8762
Extracellular vesicle	0.8003	0.6335	0. 8350	0.8574	**0.9399**
Microvesicle	0.5099	0.8974	0.6757	**0.9502**	0.9067
Mitochondrion	0.7694	0.6758	0.8332	0.8702	**0.9269**
Average AUC	087620	0.6098	0.8049	0.9033	**0.9182**

*Note:* Bold indicates the best model.

**TABLE 2:** AUPR comparison of PMLocMSCAM with other models.

Subcellular localisation	MiRLoc	MirLocPredictor	DAmiRLocGNet	PMiSLocMF	PMLocMSCAM(ours)
Cytoplasm	0.7258	0.8391	0.7636	0.8192	**0.8818**
Exosome	0.9892	0.8248	0.9248	**0.9905**	0.9675
Nucleolus	0.4148	0.4925	0.5739	0.5298	**0.6506**
Nucleus	0.8102	0.4349	0.7961	0.8763	**0.9043**
Extracellular vesicle	0.2916	0.3434	0.4619	0.4695	**0.7610**
Microvesicle	0.9203	0.2469	0.8883	**0.9866**	0.9575
Mitochondrion	0.5277	0.3113	0.6882	0.7294	**0.8188**
Average AUC	0.6689	0.4990	0.7281	0.7716	**0.8487**

*Note:* Bold indicates the best model.

Table 2 compares the AUPR metrics of different models, further validating the relatively better performance of PMLocMSCAM. In most subcellular localisation categories, PMLocMSCAM consistently outperforms PMiSLocMF. For example, in the Nucleolus category, PMLocMSCAM achieves an AUPR of 0.6506, compared to PMiSLocMF's 0.5298. This improvement is largely due to PMLocMSCAM's ability to effectively address long‐tail distributions through the use of contrastive learning loss, enhancing sensitivity to miRNA subcellular localisation categories. A particularly noteworthy result is observed in the Extracellular vesicle category, where PMLocMSCAM attains a significantly higher AUPR of 0.7610 compared to 0.4695 for PMiSLocMF. PMLocMSCAM leverages a multi‐modal data fusion framework that effectively integrates sequence‐based features, functional annotations, and interaction network information. This enables the model to capture subtle and discriminative biological signals that are often overlooked by previous models with limited data representations. Our model achieves an average AUPR of 0.8487, clearly outperforming PMiSLocMF's 0.7716. These findings highlight not only the model's superior accuracy and sensitivity but also its enhanced capability in integrating heterogeneous biological data. Such effective data fusion contributes significantly to its robust performance across complex multi‐label classification tasks in subcellular localisation.

### 4.2 Independent Dataset Test

To further assess the real‐world applicability, generalisation capability, and robustness of our proposed model, PMLocMSCAM, we constructed an independent test dataset by randomly selecting 50 miRNA samples from the original dataset. To ensure the objectivity and reliability of the evaluation, we strictly followed standardised data preprocessing procedures, including thorough data cleaning and normalisation. This guarantees that the independent test set remains completely isolated from the training set in terms of both sample origin and feature distribution, thereby effectively preventing any potential data leakage.

**FIGURE 4:**
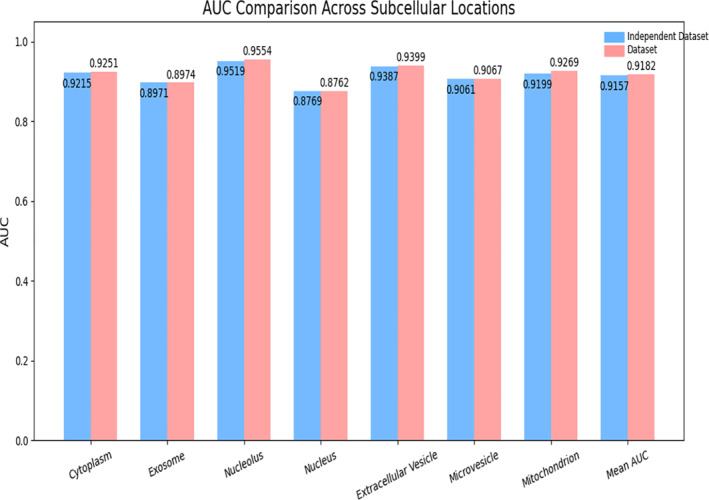
The AUC value of the model PMLocMSCAM.

**FIGURE 5:**
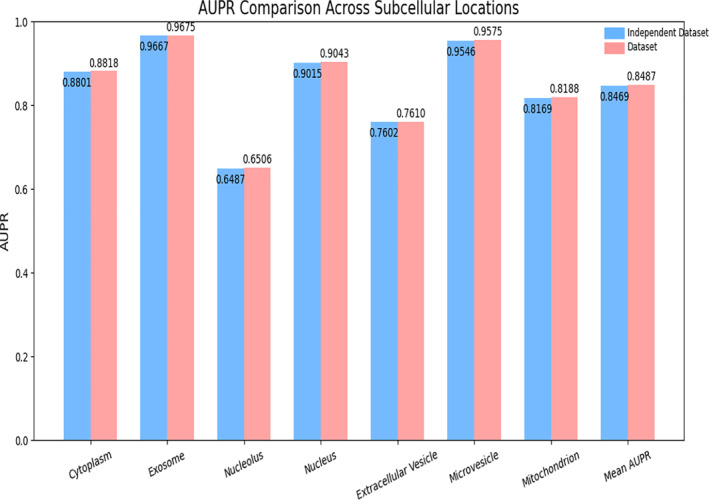
The AUPR value of the model PMLocMSCAM.

As illustrated in Figures 4 and 5, PMLocMSCAM achieves consistently high performance on the independent dataset across all subcellular locations. Specifically, the AUC values across different locations remain stable and close to those obtained on the original dataset, with a mean AUC of 0.9157. Similarly, the AUPR performance remains robust, with a mean AUPR of 0.8469. These results highlight the model's ability to capture subtle distinctions and underlying structural patterns in miRNA subcellular localisation, even under unseen test conditions.

### 4.3 Ablation Experiments and Parameter Analysis

**FIGURE 6:**
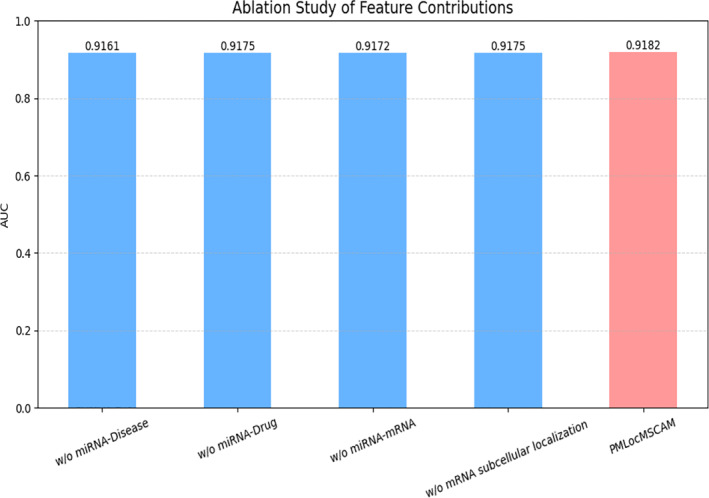
The PMLocMSCAM ablation experiment.

In addition, to systematically investigate the contribution of key architectural components—namely the Hypergraph Convolution and the Cross‐Attention Mechanism—we conducted a series of ablation and comparative experiments. As shown in Table 3, we developed several model variants by selectively removing the hypergraph convolution module, the cross‐attention module, or both, and evaluated their performance against the full PMLocMSCAM model. The experimental findings reveal that the hypergraph convolution module significantly enhances the representation of high‐order relationships among heterogeneous data sources, while the cross‐attention mechanism enables effective fusion and dynamic selection of information across different modalities. As illustrated in Figure 6, removing either module results in noticeable performance degradation across AUC, particularly under complex or incomplete data conditions. These observations underscore the synergistic effect and essential role of both modules in achieving accurate, robust, and generalisable miRNA localisation predictions.

**TABLE 3:** The Model PMLocMSCAM ablation experiment.

Subcellular localisation	Without hypergraph convolution module	Without the cross‐attention module	PMLocMSCAM
Cytoplasm	0.9149	0.9204	0.9251
Exosome	0.8765	0.8961	0.8974
Nucleolus	0.9541	0.9543	0.9554
Nucleus	0.8749	0.8760	0.8762
Extracellular vesicle	0.9381	0.9390	0.9399
Microvesicle	0.9058	0.9055	0.9067
Mitochondrion	0.9247	0.9218	0.9269
Average AUC	0.9127	0.9161	0.9182

To further investigate the practical contributions of various input features within our model, we conducted a feature ablation study by systematically removing specific categories of information and observing the resulting performance changes. Specifically, we removed the following feature types: miRNA–disease associations, miRNA–drug associations, miRNA–mRNA associations, and mRNA subcellular localisation features. The results indicate that all four feature types contribute positively to the overall predictive performance of the model, albeit to varying degrees. Notably, the removal of miRNA–mRNA interaction features led to the most significant performance degradation. This underscores the critical role of regulatory information between miRNAs and their target genes in determining subcellular localisation. Likewise, excluding mRNA subcellular localisation features also resulted in a marked performance drop, suggesting that this type of structural location information provides strong spatial guidance that enhances localisation prediction. Although the miRNA–disease and miRNA–drug features represent nonstructural semantic associations rather than spatial or molecular interaction data, their removal also caused noticeable fluctuations in model performance. This indicates that the model effectively leverages these biomedical knowledge sources to improve predictive accuracy. Collectively, these ablation experiments validate the complementarity and necessity of incorporating multi‐source heterogeneous information within the PMLocMSCAM framework. Moreover, the findings reinforce the soundness of our feature engineering strategy, which emphasises a hybrid representation that integrates both structural information and semantic relationships to capture the complex biological context of miRNA localisation.

**FIGURE 7:**
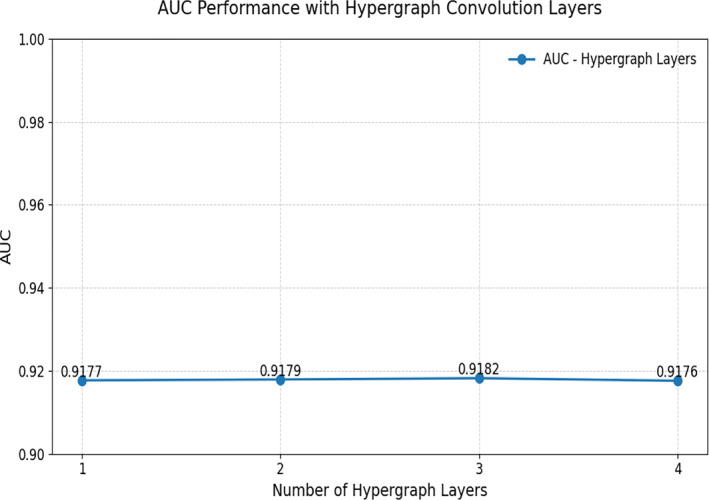
The PMLocMSCAM parameter experiments.

The experiment demonstrates the impact of the number of hypergraph convolution layers on the model's AUC performance. From Figure 7, we can observe that as the number of layers increases, the model's AUC improves slightly from 1 to 3 layers, but decreases slightly at 4 layers. Specifically, the model achieved the best AUC value of 0.9182 at 3 layers, indicating that the hypergraph convolution is most effective at capturing higher‐order structural information at this layer count. At 1 and 2 layers, the performance is slightly lower, with AUC values of 0.9177 and 0.9179, respectively. At 4 layers, although the AUC remains relatively high at 0.9176, it shows a slight decline compared to 3 layers, suggesting that an excessive number of layers may lead to overfitting, which negatively impacts the model's performance. These results indicate that in the hypergraph convolution model, an optimal layer configuration can improve performance, but too many layers do not necessarily lead to better results and may even hinder the model's predictive ability. Through this parameter experiment, we identified the optimal setting of 3 layers, which ensures efficient information propagation and learning across different layers of the model.

## 5 Case Studies

To further assess the biological reliability of our model's predictions, we conduct case studies on seven subcellular localisation types: Cytoplasm, Exosome, Nucleolus, Nucleus, Extracellular vesicle, Microvesicle, and Mitochondrion. For each compartment, we select the top 10 miRNAs with the highest predicted probabilities generated by PMLocMSCAM. Their subcellular localisations are manually verified using evidence from the scientific literature. As summarised in Table 4, we manually review the biomedical literature to verify whether these miRNAs have been experimentally confirmed to localise in the predicted categories.

**TABLE 4:** Case studies of miRNA subcellular localisations.

Rank	miRNA	Localisation	Evidence	Rank	miRNA	Localisation	Evidence
1	miR‐21‐5p	Cytoplasm	PMID:34073601	6	miR‐146b‐5p	Nucleus	PMID:28212323
2	miR‐24‐5p	Cytoplasm	PMID:38631055	7	miR‐22	Nucleus	Unconfirmed
3	miR‐375	Cytoplasm	PMID:21953699	8	miR‐210‐3p	Nucleus	PMID:30006350
4	miR‐16‐5p	Cytoplasm	PMID:33568163	9	miR‐223	Nucleus	PMID:24886830
5	miR‐146b‐5p	Cytoplasm	PMID:37108595	10	miR‐107	Nucleus	PMID:28212323
6	miR‐221‐5p	Cytoplasm	PMID:38630389	1	miR‐16‐5p	Extracellular vesicle	PMID:33568163
7	miR‐125b‐5p	Cytoplasm	PMID:38630389	2	miR‐21‐5p	Extracellular vesicle	PMID:34073601
8	miR‐181a‐5p	Cytoplasm	PMID:26975323	3	miR‐2277‐3p	Extracellular vesicle	PMID:37764278
9	miR‐107	Cytoplasm	PMID:38630389	4	miR‐21‐5p	Extracellular vesicle	PMID:34073601
10	miR‐23a‐5p	Cytoplasm	PMID:31570693	5	miR‐141‐3p	Extracellular vesicle	PMID:32034654
1	miR‐21‐5p	Exosome	PMID:34073601	6	miR‐16	Extracellular vesicle	Unconfirmed
2	miR‐122	Exosome	PMID:31732639	7	miR‐320a	Extracellular vesicle	PMID:34194551
3	miR‐149	Exosome	PMID:31732639	8	miR‐20b‐5p	Extracellular vesicle	PMID:34321587
4	miR‐21	Exosome	PMID:26674922	9	miR‐30e‐3p	Extracellular vesicle	PMID:32034654
5	miR‐140	Exosome	Unconfirmed	10	miR‐203a‐3p	Extracellular vesicle	PMID:38724995
6	miR‐150	Exosome	PMID:26674922	1	miR‐132	Microvesicle	PMID:38113807
7	miR‐320	Exosome	PMID:26674922	2	miR‐557	Microvesicle	PMID:35417986
8	miR‐203	Exosome	Unconfirmed	3	miR‐218	Microvesicle	PMID:38113807
9	miR‐30e‐3p	Exosome	PMID:26674922	4	miR‐223‐3p	Microvesicle	PMID:25938081
10	miR‐203	Exosome	PMID:26674922	5	miR‐150‐5p	Microvesicle	PMID:29284949
1	miR‐574‐3p	Nucleolus	PMID:23922894	6	miR‐126‐3p	Microvesicle	PMID:24892710
2	miR‐206	Nucleolus	PMID:17135348	7	miR‐21‐5p	Microvesicle	PMID:34073601
3	miR‐21	Nucleolus	PMID:19628621	8	miR‐16‐5p	Microvesicle	PMID:33568163
4	miR‐29b	Nucleolus	PMID:30006350	9	miR‐92a‐3p	Microvesicle	PMID:30457642
5	miR‐484	Nucleolus	PMID:23874676	10	miR‐221‐3p	Microvesicle	Unconfirmed
6	miR‐193b	Nucleolus	PMID:23922894	1	miR‐26b‐5p	Mitochondrion	PMID:36466236
7	miR‐1	Nucleolus	PMID:19628621	2	miR‐21‐5p	Mitochondrion	PMID:34073601
8	miR‐125b	Nucleolus	Unconfirmed	3	miR‐155‐5p	Mitochondrion	PMID:27551467
9	miR‐351	Nucleolus	PMID:19628621	4	miR‐96‐5p	Mitochondrion	PMID:29749455
10	miR‐191	Nucleolus	PMID:23874676	5	miR‐338‐3p	Mitochondrion	PMID:27265729
1	miR‐21‐5p	Nucleus	PMID:34073601	6	miR‐365	Mitochondrion	PMID:19941672
2	miR‐24‐5p	Nucleus	PMID:32883147	7	miR‐302a	Mitochondrion	PMID:19941672
3	miR‐67	Nucleus	Unconfirmed	8	miR‐378	Mitochondrion	PMID:32324435
4	miR‐21	Nucleus	PMID:19628621	9	miR‐34a	Mitochondrion	Unconfirmed
5	miR‐122	Nucleus	PMID:17135348	10	miR‐1	Mitochondrion	PMID:32324435

As shown in Table 4, our model predicted 70 miRNA subcellular localisation associations, of which 62 are supported by relevant literature, while only 8 predictions cannot be validated. Based on these results, we selected several representative miRNAs for detailed case studies. MiR‐21‐5p is one of the most widely studied oncogenic miRNAs, with experimental evidence confirming its predominant localisation in the cytoplasm, where it promotes tumour progression by repressing PDCD4 and PTEN expression [40]. miR‐21‐5p has also been detected in the exosome, microvesicle, and mitochondrion [41], highlighting its potential role in intercellular signalling and organelle‐level regulation. Our model accurately predicted the localisation of miR‐21‐5p in the cytoplasm, extracellular vesicle, and mitochondrion, where it consistently received top scores. Similarly, miR‐16‐5p is a well‐characterised tumour‐suppressive miRNA involved in the post‐transcriptional regulation of BCL2 and Wnt/β‐catenin signalling [42, 43]. This miRNA has been validated in both the cytoplasm and extracellular vesicle, and our model precisely reflected this, supporting its practical value in identifying functionally relevant localisation patterns. miR‐122 is a liver‐specific miRNA essential for lipid metabolism and hepatitis C virus replication, experimentally localised in the nucleus and exosome [44]. Our model successfully identified these two categories as top‐ranked predictions, consistent with its dual regulatory functions. miR‐107 is known for its role in neuronal development and tumour metabolism and has been reported in both the nucleus and cytoplasm [45, 46]; our predictions matched this distribution, indicating accurate modelling of multi‐class miRNAs. miR‐21 (closely related to miR‐21‐5p but treated separately in some datasets) has been observed in the nucleus, nucleolus, and exosome [47]. The 8 unconfirmed miRNAs deserve further in‐depth experimental exploration and validation in the future. These findings indicate that PMLocMSCAM not only achieves overall predictive performance but also generates biologically meaningful insights, which may guide future experimental validations.

## 6 Conclusion

Recently, miRNAs have become the important new ideas for human complicated disease diagnosis and treatment. Subcellular localisation of miRNAs is a key reflection of their biological functions. However, miRNAs have specific physiological roles when they are at different cellular sites. Therefore, in order to systemically understand the physiological function of miRNAs, identifying the subcellular localisation has become a hot topic in miRNA current research filed. By considering the limits of wet‐experiment being time‐consuming and expensive, some computational methods have proposed to predict miRNA subcellular localisation.

In this study, we also provide a computational method to identifying miRNA subcellular localisation. PMLocMSCAM integrates multimodal miRNA data (sequence, disease/drug/mRNA associations) through a streamlined computational workflow. Initially, sequence similarity and functional features are derived via Smith–Waterman alignment and disease ontology analysis. Three interaction networks (miRNA‐disease, miRNA‐drug, miRNA‐mRNA) are processed by Node2vec for topological embedding, combined with hypergraph convolution for high‐order relationship extraction. miRNA‐mRNA localisation signals are weighted through interaction networks, followed by multi‐head attention fusion of five core features: sequence, disease association, drug association, mRNA interaction, and localisation patterns. Residual‐connected MLPs subsequently generate probabilistic predictions across seven subcellular compartments, establishing an end‐to‐end multimodal prediction framework.

However, although our method obtains a good result in identifying miRNA subcellular localisation, there are still left some limits to improve. First, by considering miRNAs are short RNA species derived from hairpin‐forming miRNA precursors (pre‐miRNA), the pre‐miRNA pre‐miRNA sequence information also should be take part into calculating miRNA sequence similarity. Second, the new GNN model also should be considered to more effectively extract miRNA feature from association network. Therefore, based on the importance of predicting miRNA subcellular localisation and strong motivation for efficient computational method, we would propose new miRNA subcellular localisation method to address these limits in the future.

## Author Contributions

**Jipu Jiang:** data curation, formal analysis, methodology, software, validation, visualization, writing – original draft, writing – review and editing. **Cheng Yan:** conceptualization, funding acquisition, investigation, methodology, project administration, supervision, writing – review and editing.

## Conflicts of Interest

The authors declare no conflicts of interest.

## Data Availability

The original data presented in the study are openly available in the public domain: RNALocate v2.0 at http://www.rnalocate.org/ or http://www.rna‐society.org/rnalocate/. The code and data that support the findings of this study is available at https://github.com/27167199/PMLocMSCAM.
